# Alterations of human CSF and serum-based mitophagy biomarkers in the continuum of Alzheimer disease

**DOI:** 10.1080/15548627.2024.2340408

**Published:** 2024-05-02

**Authors:** Kateřina Veverová, Jan Laczó, Alžběta Katonová, Hana Horáková, Veronika Matušková, Francesco Angelucci, Martina Laczó, Zuzana Nedelská, Jakub Hort, He-Ling Wang, Jianying Zhang, Liu Shi, Evandro Fei Fang, Martin Vyhnálek

**Affiliations:** a Memory Clinic, Department of Neurology, Second Faculty of Medicine, Charles University and Motol University Hospital, Prague, Czech Republic; b Department of Clinical Molecular Biology, University of Oslo and Akershus University Hospital, Lørenskog, Norway; c Department of Psychiatry, University of Oxford, Oxford, UK; d The Norwegian Centre on Healthy Ageing (NO-Age), Oslo, Norway

**Keywords:** Autophagy, BNIP3L, mild cognitive impairment, mitophagy, PINK1, TFEB

## Abstract

Defective mitophagy is consistently found in postmortem brain and iPSC-derived neurons from Alzheimer disease (AD) patients. However, there is a lack of extensive examination of mitophagy status in serum or cerebrospinal fluid (CSF), and the clinical potential of mitophagy biomarkers has not been tested. We quantified biomarkers of mitophagy/autophagy and lysosomal degradation (PINK1, BNIP3L and TFEB) in CSF and serum from 246 individuals, covering mild cognitive impairment due to AD (MCI-AD, *n* = 100), dementia due to AD (AD-dementia, *n* = 100), and cognitively unimpaired individuals (CU, *n* = 46), recruited from the Czech Brain Aging Study. Cognitive function and brain atrophy were also assessed. Our data show that serum and CSF PINK1 and serum BNIP3L were higher, and serum TFEB was lower in individuals with AD than in corresponding CU individuals. Additionally, the magnitude of mitophagy impairment correlated with the severity of clinical indicators in AD patients. Specifically, levels of PINK1 positively correlated with phosphorylated (p)-MAPT/tau (181), total (t)-MAPT/tau, NEFL (neurofilament light chain), and NRGN (neurogranin) levels in CSF and negatively with memory, executive function, and language domain. Serum TFEB levels negatively correlated with NEFL and positively with executive function and language. This study reveals mitophagy impairment reflected in biofluid biomarkers of individuals with AD and associated with more advanced AD pathology.

**Abbreviation:** Aβ: amyloid beta; AD: Alzheimer disease; AVs: autophagic vacuoles; BNIP3L: BCL2 interacting protein 3 like; CU: cognitively unimpaired; CSF: cerebrospinal fluid; LAMP1: lysosomal-associated membrane protein 1; MAP1LC3/LC3: microtubule associated protein 1 light chain 3; MCI: mild cognitive impairment; NRGN: neurogranin; NEFL: neurofilament light chain; p-MAPT/tau: phosphorylated microtubule associated protein tau; PINK1: PTEN induced kinase 1; t-MAPT/tau: total microtubule associated protein tau; TFEB: transcription factor EB; TMT: Trail Making Test.

## Introduction

Alzheimer disease (AD) is the leading cause of neurocognitive disorder in adults, affecting more than 50 million individuals worldwide [1,2]. Lecanemab and donanemab, antibodies to β-amyloid (Aβ) developed for pharmacotherapeutic use in AD patients, represent one of the very few promising approaches for managing early stages of AD. While it is exciting that these drugs reduced the rate of AD-associated memory loss by approximately 35% during phase 3 clinical trials, it is unfortunate that drugs or strategies that cure AD-associated cognitive decline have not yet been discovered [3,4]. To overcome the roadblocks and knowledge gaps that hinder the development of such therapies, an improved mechanistic understanding of AD is urgently needed. Another need is for clinically useful diagnostic, therapeutic, and/or prognostic biomarkers of AD [5].

Macroautophagy/autophagy is a cellular self “garbage clearance” system through which cells eliminate and recycle damaged and dysfunctional cytoplasmic components, including organelles, misfolded protein aggregates, among others [6,7]. Mitophagy is a subtype of autophagy that recognizes and degrades damaged or superfluous mitochondria. Autophagy and mitophagy are required to maintain cellular and mitochondrial homeostasis, respectively; they also maintain energy balance and cell signaling processes that ensure cellular resilience and survival [8–10]. Our previous studies in postmortem brain from AD patients showed that the rate of mitophagy decreases with patient age and that capacity for mitophagy is lower in AD patient-derived iPSC neurons. Data from several animal model support the idea that reduced mitophagy is associated with increased AD risk and pathophysiology [11,12]. Autophagy and mitophagy are impaired in AD as evidenced by reduced autophagy/mitophagy event (using electron microscopy techniques), low colocalization of the mitochondrial protein TOMM20 to the lysosomal protein LAMP2, and altered levels/activities of key autophagy/mitophagy proteins such as ULK1, AMPK, PICALM, BECN1, PI3P, AMBRA1, TBK1, BCL2L13, PINK1, BNIP3L/NIX, DISC1, BNIP3, PS1, CTSB (cathepsin B), and CTSD [6,12–15]. Reduced mitophagy is likely a “culprit”, but not a “bystander” of AD, as pharmacological (NAD^+^ precursors, urolithin A, actinonin, kaempferol, rhapontigenin [11,12]) and genetic (e.g., overexpression of PINK1 [16]) upregulation of mitophagy restore mitochondrial homeostasis, abrogated AD-related pathology, and reduced memory loss in animal models of AD. The latter results are consistent with the idea that reduced mitophagy is a critical determinant of AD development and suggest that the levels of mitophagy proteins could have potential as biomarkers of AD progression and/or diagnosis. In the context of developing new interventions targeting mitochondrial function, we wanted to investigate whether key substances involved in mitophagy are altered in AD and whether they can be detected in body fluids [17]. Additionally, we wanted to explore the feasibility of developing mitophagy biomarkers for clinical AD assessment.

To this end, we pursued the following three aims. First, we quantified mitophagy biomarkers in cerebrospinal fluid (CSF) and serum from biomarker-defined individuals in various stages of AD and cognitively unimpaired (CU) individuals and determined whether mitophagy biomarker levels correlated with AD stage. Also, we explored whether these changes are influenced by a major genetic risk factor for sporadic AD, *APOE* (apolipoprotein E) ɛ4. Second, it was determined whether quantitative clinical biomarkers of AD pathology (*i.e.*, amyloid beta 42 [Aβ42], Aβ42/40, phosphorylated MAPT/tau (181) (p-MAPT/tau [181]) and total MAPT/tau (t-MAPT/tau) neurodegeneration (NEFL [neurofilament light chain]), synaptic dysfunction (NRGN [neurogranin]), cognitive status and brain atrophy correlated with biomarkers of mitophagy. Third, it was determined whether mitophagy biomarkers in serum and CSF correlated with each other. We included biomarkers of various mitophagy steps: PINK1 (PTEN induced kinase 1; a mitochondrial kinase crucial for mitophagy activation), BNIP3L (BCL2 interacting protein 3 like; a mitophagy receptor), and TFEB (transcription factor EB; a master regulator of lysosome biogenesis and autophagy) [6,10].

Our results demonstrated that mitophagy markers in CSF and serum are changed in AD continuum, mitophagy activators PINK1 and BNIP3L increased while TFEB decreased, indicating the impairment in the final step of autophagy-lysosomal degradation. Furthermore, mitophagy biomarkers were related to the severity of AD pathology.

To the best of our knowledge, this is the first study to evaluate quantitative measures of mitophagy in human CSF and serum in the prodromal stages of AD.

## Results

### Changes of mitophagy proteins in CSF and serum from CU, AD-MCI, and AD dementia

Using commercially available ELISA kits, PINK1 was detected in CSF, but BNIP3L and TFEB were not. This could reflect that the antigen concentration in CSF was below the level of detection (tested with all commercially available kits). PINK1, BNIP3L, and TFEB were detected in normal serum using commercially available ELISA kits.

**Figure 1.**
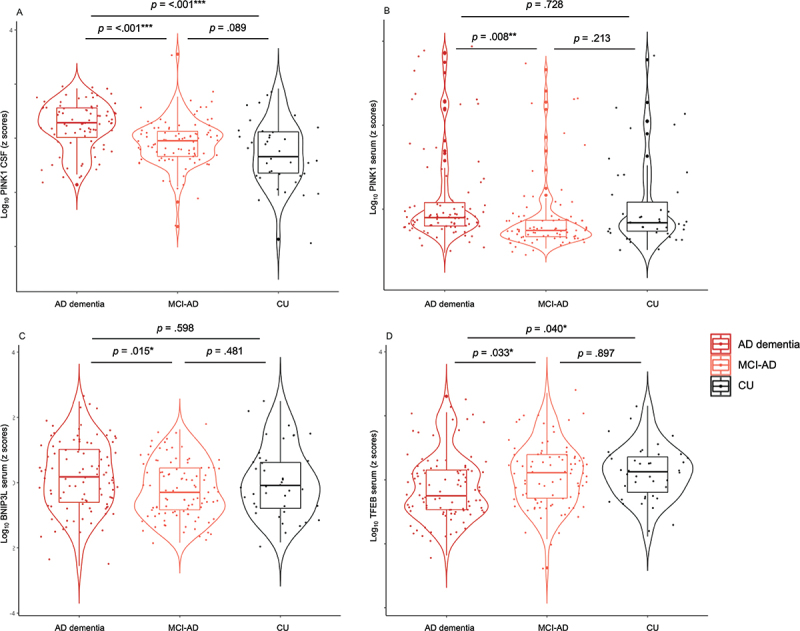
Changes in mitophagy biomarkers across AD continuum. Violin plots of ANCOVA analyzes (A–D) levels of mitophagy markers PINK1, BNIP3L, and TFEB in biomarker-defined individuals. (A) CSF PINK1 showed higher levels in AD dementia compared to MCI-AD and CU groups. (B) Serum PINK1 showed higher levels in AD dementia compared to MCI-AD group. (C) Serum BNIP3L showed higher levels in AD dementia compared to MCI-AD group. (D) Serum TFEB showed lower levels in AD dementia compared to MCI-AD and CU groups. Notes: Data were adjusted for sex and age. *** = p < .001, ** = p < .01, * = p < .05. Abbreviations: AD, Alzheimer disease; MCI, mild cognitive impairment; CSF, cerebrospinal fluid; CU, cognitively unimpaired.

In CSF, the levels of PINK1 differed significantly among patient subgroups (F [2] = 19.92, *p* = <0.001, η^2^ = 0.14): More specifically, PINK1 was significantly higher in AD dementia individuals (1.3 ± 0.2 ng/mL) than in MCI-AD (1.1 ± 0.2 ng/mL) (*p* < .001) and CU (1.0 ± 0.3 ng/mL) (*p* < .001) (Figure 1A). Similar results were observed in serum (F [2] = 4.57, *p* = 0.012 η^2^ = 0.04) with higher PINK1 in AD dementia individuals (6.4 ± 13.3 ng/mL) than in MCI-AD (3.2 ± 6.8 ng/mL) (*p* = 0.008) (Figure 1B). The levels of BNIP3L in serum also differed between study subgroups (F [2] = 3.96, *p* = 0.020, η^2^ = 0.03) with significantly higher BNIP3L in AD dementia individuals (2.9 ± 2.1 ng/mL) than in MCI-AD (2.1 ± 1.2 ng/mL) (*p* = 0.015) (Figure 1C). In contrast, serum levels of TFEB were significantly lower in AD dementia individuals (670.1 ± 368.6 pg/mL) than in MCI-AD (744.3 ± 351.9 pg/mL) (*p* = 0.033) and CU (966.5 ± 1562.5 pg/mL) (*p* = 0.040) (F [2] = 6.20, *p* = 0.002, η^2^ = 0.04) (Figure 1D). These data are summarized in Table 2.

To evaluate the genetic contribution of the *APOE* ε4 allele on the differences of these three proteins between AD and controls, we added the *APOE* genotype into the ANCOVA analysis as a covariate (*APOE* e4+ vs e4-). This step did not substantially change the results between mitophagy markers and the study cohorts. There were still significant differences among patient subgroup with the levels of CSF PINK1 (F [2] = 20.34, *p* = <0.001, η^2^ = 0.12), levels of serum PINK1 (F [2] = 4.56, *p* = 0.012, η^2^ = 0.04), levels of serum BNIP3L (F [2] = 4.50, *p* = 0.012, η^2^ = 0.03) and levels of serum TFEB (F [2] = 6.30, *p* = 0.002, η^2^ = 0.05).

To assess the contribution of neurodegeneration to the differences in CSF PINK1 levels between AD and controls, we included CSF levels of neurofilament light chain (NEFL), a biomarker of neurodegeneration, as a covariate in the ANCOVA analyzes. This step did not substantially change the results between the mitophagy markers and the study cohorts: particularly the effect sizes remained unchanged and the differences among patient subgroups were still significant differences with the levels of CSF PINK1 levels (F [2] = 20.55, *p* = <0.001, η^2^ = 0.14), serum PINK1 levels (F [2] = 4.68, *p* = 0.011, η^2^ = 0.04), serum BNIP3L levels (F [2] = 5.71, *p* = 0.004, η^2^ = 0.04) and serum TFEB levels (F [2] = 4.338 *p* = 0.014, η^2^ = 0.04).

### Correlation of mitophagy markers (PINK1, BNIP3L, and TFEB) with AD biomarkers (Aβ42/40, Aβ42, p-MAPT/tau [181], t-MAPT/tau, NEFL, NRGN), AT(N) framework, cognitive status, and AD-related brain structures

**Figure 2.**
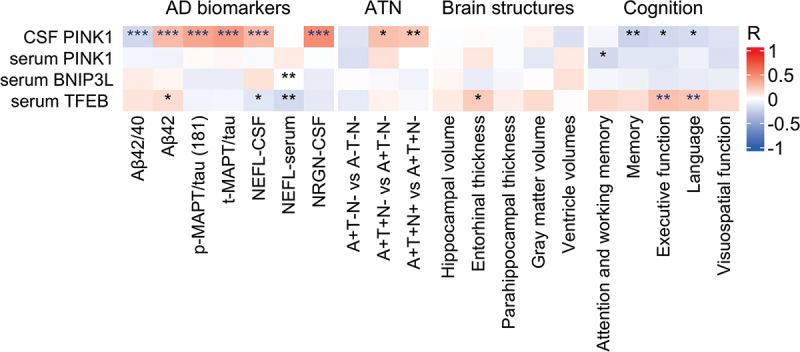
Mitophagy biomarkers vs Alzheimer disease (AD) phenotype. *, ** and *** denote *p* < 0.05, *p* < 0.01 and *p* < 0.001, respectively. Partial Pearson correlation for AD biomarkers, ATN and brain structures was adjusted for age and sex or in the case of cognition, for age, sex, and education. Blue asterisks denote significance after Holm-Bonferroni correction for multiple comparisons. CSF, cerebrospinal fluid; “A”, amyloid; “T”, tau; “N”, neurodegeneration; +, abnormal; -, normal; Aβ42/42, amyloid beta 42 to 40 ratio; Aβ42, amyloid beta 42; *p*-MAPT/tau (181), phosphorylated MAPT/tau (181); *t*-MAPT/tau, total MAPT/tau; NEFL, neurofilament light chain; NRGN, neurogranin.

The data were analyzed to determine associations between mitophagy biomarkers (PINK1, BNIP3L, TFEB), AD biomarkers (Aβ42/40, Aβ42, p-MAPT/tau [181], t-MAPT/tau), and markers of neurodegeneration and synaptic dysfunction (NEFL and NRGN, respectively). Weak to moderate correlations were found between PINK1 in CSF and AD biomarkers in CSF. More specifically, PINK1 correlated negatively with Aβ42/40 (*r* = −0.214, *p* < .001), and positively with Aβ42 (*r* = 0.285, *p* < .001), p-MAPT/tau (181) (*r* = 0.381, *p* < .001), and t-MAPT/tau (*r* = 0.423, *p* < .001). PINK1 also exhibited moderate positive correlation with NEFL (*r* = 0.294, *p* < .001) and NRGN (*r* = 0.490, *p* < .001) in CSF. Serum BNIP3L correlated positively with serum NEFL (*r* = 0.181, *p* = 0.004). Serum TFEB correlated negatively with NEFL in CSF (*r* = −0.132, *p* = 0.038) and serum (*r* = −0.202, *p* = 0.001) (Figure 2).

The severity of AD pathology was evaluated using the AT(N) framework, and these data were analyzed for relationships with PINK1, BNIP3L, and TFEB. The results revealed weak positive correlation between PINK1 in CSF and A+T+N- vs. A+T-N- (*r* = 0.274, *p* = 0.028) and A+T+N+vs. A+T+N- (*r* = 0.238, *p* = 0.009) (Figure 2). In addition, PINK1 in CSF showed weak negative correlation with a memory domain (*r* = −0.242, *p* = 0.007), executive function (*r* = −0.206, *p* = 0.024), and a language domain (*r* = −0.186, *p* = 0.038). Serum PINK1 showed similar weak negative correlation with attention and working memory (*r* = −0.193, *p* = 0.034). In contrast, serum TFEB correlated positively with executive function (*r* = 0.266, *p* = 0.002), a language domain (*r* = 0.241, *p* = 0.006) (Figure 2), and entorhinal thickness (*r* = 0.221, *p* = 0.014). No other correlations were observed between mitophagy biomarkers in CSF or serum and AD-related brain structural change (Figure 2).

### Correlation between mitophagy markers (PINK1, BNIP3L, and TFEB) in all study groups

**Figure 3.**
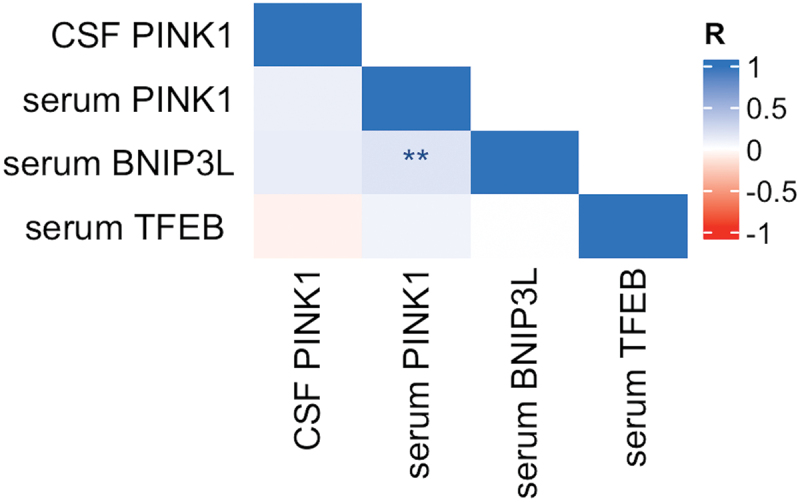
Correlation between mitophagy biomarkers in serum and CSF. *Notes*: Pearson correlation, *** = *p* < .001, ** = *p* < .01, * = *p* < .05. *Abbreviations*: CSF, cerebrospinal fluid.

Levels of PINK1 in CSF did not correlate with levels of PINK1 in serum (*r* = 0.106, *p* = 0.162). PINK1 in serum correlated positively with BNIP3L in serum (*r* = 0.185, *p* = 0.008). No other correlations between mitophagy markers were detected (Figure 3).

## Discussion

We investigated the alteration of mitophagy markers among patients with AD (MCI-AD and AD dementia) and CU individuals. Our data from 246 biomarker-defined individuals show a significant change in levels of CSF and serum-based mitophagy biomarkers in the AD continuum. This study reveals that mitophagy activators PINK1 in CSF and serum and BNIP3L in serum are higher in AD dementia individuals than in MCI-AD and CU controls, while serum TFEB, a master regulatory of lysosomal biogenesis, is lower in AD dementia than in MCI-AD individuals and CU controls independently of *APOE* status or levels of neurodegeneration. These findings are consistent with the hypothesis that mitophagy defects contribute to AD pathology and progression and suggest the impairment in the final stage of autophagy – lysosomal degradation.

Historically, the first evidence that autophagy plays a role in protein homeostasis and surveillance in the human brain was the presence of highly abundant autophagosomes and prelysosomal autophagic vacuoles (AVs) in neocortical and hippocampal pyramidal neurons and dystrophic neurites (including synaptic terminals) of AD patients, while similar pathology was absent from brains of normal control individuals [18]. Subsequently, it was shown that AVs in brain tissue and protein markers of AVs such as MAP1LC3/LC3 (microtubule associated protein 1 light chain 3)-II increase with increasing AD progression/severity, being higher in later Braak stage than in early Braak stage AD patients and normal controls [19].

Evidence provided here supports the idea that mitophagy plays a role in AD pathology, demonstrating that PINK1 in CSF and serum and BNIP3L in serum increase with advanced AD disease severity. Earlier studies report similar observations: upregulated levels of mitophagy activators, PINK1 and PRKN, were also found in the hippocampal biopsies of patients with sporadic AD and were accompanied by abnormally increased mitochondrial content [20]. Additionally, a similar alteration was found in the fibroblasts of the same patients suggesting an impaired mitochondrial function in peripheral tissue [20] as well as accumulation of AVs containing abnormal mitochondria [21], suggesting reduced mitophagic flux in cells from AD patients. Increased levels of PRKN and “mitophagy tag” p-S65-Ub levels in brain biopsies from patients with AD compared to controls also suggest the impairment in the final degradation step of mitophagy [22].

It is important to note, that several studies of human brain biopsies have found reduced PINK1 levels in brains with AD pathology [16,23,24] For example, Vaillant-Beuchot and his team described decreased levels of PINK1 in Alzheimer brain biopsies compared to control samples, but in the same paper, they presented increased levels of PINK1 in APPswe cells compared to control cells [24]. Brain samples showed elevated levels of LC3-I/II (autophagosome markers), which, similar to our work, was interpreted as intracellular autophagosome accumulation and dysfunctional lysosomal degradation [18]. Likewise, a comprehensive study of hippocampal CA1 pyramidal neurons also demonstrated that autophagy increases in early-stage AD (Braak stage III) and subsequently decreases in late-stage (Braak stage V), reflected by the accumulation of LC3-II and SQSTM1/p61 and increased autolysosomal size and total area [25].

Interpreting results from studies that present decreased levels of PINK1 is challenging, as the control samples did not represent individuals without neurodegenerative pathology [16,23,24]. The control brain biopsies in the study by Vaillant-Beuchot et al. included six healthy controls and three patients with amyotrophic lateral sclerosis, another neurodegenerative disease that shares common neurodegenerative pathways with AD [24]. Du et al. compared AD individuals (*n* = 7) with non-AD individuals (*n* = 7), which included individuals without Aß plaques but with positive neurofibrillary tangles (Braak stage I-IV), again another neurodegenerative pathology [16]. Sohn et al. compared *APOE* ɛ4 carriers with non-carriers. The *APOE* ɛ4 non-carriers included seven individuals – six patients – three with PART, two with AD, one with ARTAG, and one normal individual. In other words, they investigated the effect of the *APOE* polymorphism on mitophagy markers in two clinical groups [23].

To date, limited data on the expression of autophagy/mitophagy markers in human CSF and blood is available. Mitophagy-associated proteins were investigated in human biofluids with other neurological diseases [26,27]. Increased PINK1 levels were found both in the serum and CSF of patients with multiple sclerosis [27] and in the plasma of individuals with Parkinson disease [26] compared to healthy controls. Regarding AD, two previous studies also found that increased levels of other protein activators of autophagy and lysosomal pathways are higher in CSF in patients compared to controls [28,29]. Specifically, EEA1 (early endosome antigen 1), RAB3, RAB7 (endosomal proteins), LAMP1 (lysosomal associated membrane protein 1) and LAMP2 (lysosomal proteins), LC3 (a marker of autophagosomes) and LAMP2 (a marker of AVs) were significantly higher in CSF of AD patients than in controls [28,29]. In addition, LAMP-2-deficient mice show accumulated AVs in many tissues (liver, pancreas, muscle, and heart) [30], suggesting that altered levels may indicate lysosomal dysfunction in AD.

Another possible explanation for the discrepancies between brain biopsy and biofluid studies is that researchers working with brain biopsies only examine certain regions of the brain [16,23,24], but the biofluid variations likely reflect the overall changes.

Cytoplasmatic accumulation of AVs and elevated levels of various autophagy/mitophagy markers have been explored to imply increased autophagy in neurons. However, the increased numbers of autophagosomes and their markers can also accumulate intracellularly (and in other biological fluids) when the final step of autophagy/mitophagy is impaired, and autophagosomes cannot fuse with lysosomes [31]. To distinguish between increased autophagic response and autophagy blockade, it was suggested to consider autophagic flux [32].

TFEB is a master regulator of proteins involved in lysosome biogenesis and function and autophagic flux [33]. Here, we show that TFEB protein is less abundant in serum of AD dementia individuals than in MCI-AD and CU individuals, which is consistent with previous studies showing lower expression of TFEB in monocytes and lymphocytes [34] and brain biopsies from individuals with AD. In the hippocampus, expression of nuclear TFEB decreased with increasing Braak-stage [35]. A study with iPCS-derived neurons from patients with familial AD demonstrated low TFEB, together with increased PINK1 and PRKN, and abundant damaged mitochondria [36]. In addition, TFEB knockdown mice show higher LC3B-I and LC3-II and more abundant autophagosomes than control mice. The lysosomal markers colocalize and co-purify with mitochondria in TFEB-deficient cells, suggesting blockage in the late stages of mitophagy [37]. Overall, these data imply that depletion of TFEB impairs autophagy/mitophagy and could play a role in AD pathology [35–37]. Conversely, some studies suggest that upregulation/overexpression of TFEB promotes degradation of Aß and tau aggregates, reduces cell apoptosis, and prevents memory impairment through autophagy-lysosome pathway in AD transgenic mice [38–40]. Little is known about TFEB expression in CSF. Only one study analyzed the TFEB levels in CSF from AD patients and controls. Even though they found elevated levels of autophagosome marker LC3, they found no differences in TFEB levels between the groups [28]. These negative results might be due to the small sample size and heterogeneous cohort. They included only 20 participants defined by abnormal AD biomarkers (Aβ42, p-MAPT/tau [181]), t-MAPT/tau) but without any clinical definition [28]. To the best of our knowledge, this is the first study to quantify TFEB in CSF and serum from well-defined AD patients stratified by clinical stages.

The findings of the present study reveal that expression of mitophagy markers is affected primarily in later stages of AD (*i.e.*, AD dementia). However, evidence from animal models of AD predict such changes in the early stages of AD (*i.e*., MCI-AD), and a study by Nixon and colleagues reported increased expression of LC3-II in postmortem brain of Braak stage I-II patients measured by western blot [19]. While additional studies are needed to resolve this discrepancy, it could indicate that changes in earlier stages of AD occur intracellularly and are released into the extracellular space in very low concentrations and fell below the level of detection. Our findings in the advanced stage of the disease may reflect neurodegeneration when the metabolites are released in extracellular space in higher concentrations and are detectable by standard ELISA measurement. However, our analyzes showed that adding CSF NEFL levels, a biomarker of neurodegeneration, as a covariate in the ANCOVA analyzes did not alter the results between the mitophagy markers and the study cohorts.

Among other results, we demonstrate a statistically significant correlation between CSF PINK1 and AD biomarkers, including a lower Aβ42/40 ratio and higher p-MAPT/tau (181), t-MAPT/tau, NEFL, and NRGN. A weak positive correlation between PINK1 and Aβ42 found in the whole sample as well as in the patient groups was unexpected but not necessarily in contradiction with our finding of the PINK reduction in clinical groups. Knowing that it is generally accepted that Aβ42 in clinical stages of AD does not reflect the clinical severity nor disease activity, we expect that other pathophysiological factors explaining the interindividual variability in Aβ42 May exist [41]. Also, more severe AD pathology, according to the AT(N) framework, correlated with higher PINK1 in CSF. Also, serum TFEB and serum BNIP3L levels were associated with the severity of AD pathology, as demonstrated by the negative correlation with NEFL (markers of neurodegeneration). Levels of CSF PINK1 did not correlate with serum PINK1, and serum PINK1 did not correlate with AD biomarkers, suggesting that PINK1 is issued mainly from peripheral tissues. However, the increased levels of PINK1 in AD also appear in serum samples, which indirectly confirms the ubiquitous nature of the mitochondrial alteration not limited to brain tissue.

AD patients typically experience cognitive impairment, especially affecting the memory domain. Data from animal studies associate defects in autophagy/mitophagy in hippocampal neurons with reduced performance in memory tasks [12,16,42], and it has been proposed that autophagy/mitophagy is crucial for memory formation [42]. Our data demonstrate a negative correlation between CSF PINK1 and memory, executive function, and a language domain and a negative correlation between serum PINK1 and attention and working memory. TFEB also correlated positively with executive function and a language domain. These findings are consistent with the hypothesis that mitophagy is required for normal cognition. In animal models of AD, it has been reported that pharmacological [11,12] or genetic [16] stimulation of mitophagy can reverse memory impairment.

Mitophagy markers were not associated with AD-related changes in brain structure in the present study, in contrast to previous reports that found brain-region-specific accumulation of mitophagy markers in AD patients’ brain tissue [19,20].

A strength of present study is the uniqueness of the patient cohort followed in CBAS. As such, all study participants underwent clinical examination, comprehensive neuropsychological assessment, brain MRI, and biomarker evaluation, and all studies were carried out at a single clinic and laboratory. A limitation of the current study is the relatively small size of the study cohort, especially the control group, which is mainly caused by the difficulty in recruiting biomarker-defined healthy elderly.

In summary, the upregulation of mitophagy activators in serum and CSF and downregulation of TFEB in serum of AD patients with dementia indicate impairment in autophagy-lysosomal pathway degradation and suggest that dysregulation of autophagy/mitophagy plays a significant role in onset and progression of AD. Our future research goals are to improve the detection of low abundance mitophagy markers in CSF and to conduct a longitudinal study of mitophagy/autophagy biomarkers in biofluids of AD patients.

## Materials and methods

### Participants

This study included 246 biomarker-defined participants recruited from the Czech Brain Aging Study (CBAS) cohort, the CBAS plus cohort at the Memory Clinic of Charles University/Motol University Hospital, and the Department of Neurology, Motol University Hospital in Prague, Czech Republic. All study participants signed an informed consent form approved by the local ethics committee (number EK218/20) [18]. All participants included in the study were White and of Czech nationality. Participants with cognitive deficit (*n* = 200) were referred to the Memory Clinic by general practitioners or neurologists for cognitive complaints reported by themselves or their informants. Cognitively unimpaired (CU) older adult participants (*n* = 46) had previously undergone lumbar puncture that was negative for pathological conditions.

All study participants were subject to clinical evaluation, including routine blood tests, cognitive assessment, brain magnetic resonance imaging (MRI) and quantitative assessment of Aβ in the brain. The majority of participants (*n* = 218) underwent lumbar puncture to donate CSF, which was analyzed for Aβ, p-MAPT/tau (181) and t-MAPT/tau; patients who did not donate CSF (*n* = 24) underwent PET imaging to assess Aβ load.

**Participants with AD dementia (*n* = 100)** met the clinical criteria for high likelihood of AD dementia, including evidence of AD pathophysiology, progressive impairment in at least two cognitive domains (*i.e*.,≥1.5 standard deviations [SD] lower memory test score than the age- and education-adjusted norms as well as similarly low score in at least one non-memory cognitive test) and significant impairment in activities of daily living [43]. The participants had CSF positive for AD biomarkers (reduced Aβ42 and elevated p-MAPT/tau [181] [<620 pg/mL and >61 pg/mL, respectively] [44]) (*n* = 93) and/or were positive for Aβ based on 18F-flutemetamol PET scan (*n* = 9). In patients who did not undergo CSF collection the neuronal injury was determined based on visual rating of the hippocampus [45].

**Participants with mild cognitive impairment due to AD (MCI-AD)** (*n* = 100) met the clinical criteria for high likelihood of aMCI due to AD including memory complaints, evidence of memory impairment (*i.e*., ≥1.5 SDs lower score than the age- and education-adjusted norms in any memory test), but largely intact activities of daily living and absence of dementia [46]. The participants had CSF positive for AD biomarkers (reduced Aβ42 and elevated p-MAPT/tau [181] [<620 pg/mL and >61 pg/mL, respectively] [44]) (*n* = 90) and/or were positive for Aβ based on PET imaging (*n* = 8). In patients who did not undergo CSF collection, the neuronal injury was determined based on visual rating of the hippocampus [45]. As expected, MCI-AD scored lower than CU participants and higher than AD dementia patients in all cognitive tests (ps < .05).

**CU participants** (*n* = 46) met one of two sets of criteria.

1. Participants referred by the Department of Neurology met the following criteria: underwent lumbar puncture to exclude inflammatory disorder (*e.g*., facial palsy, headache, back pain), were negative for pathological and inflammatory markers in CSF and blood (*n* = 39), did not report cognitive complaints, demonstrated cognitive performance within the age- and education-adjusted normal range. In addition, they had no evidence of hippocampal atrophy on MRI and had normal results of AD biomarkers in CSF.
2. among the patients followed in the CBAS study for subjective cognitive decline (SCD) (*n* = 7). These SCD controls reported cognitive complaints that motivated them to seek medical help, but they did not have any impairment in activities of daily living and demonstrated cognitive performance within the normal age- and education-adjusted normal range [47]. In addition, they had no evidence of hippocampal atrophy on MRI and had normal results of AD biomarkers in CSF or on PET.

Demographic data and prevalence of the most common comorbidities are shown in Table 1.

**Table 2.** Mitophagy and AD biomarkers in CSF and serum.

	Memory Clinic Cohort				P-values		
	All patients	AD Dementia	AD-MCI Patients	CU Controls	AD-MCI vs. ADDementia	AD MCIvs. CU	AD Dementiavs. CU
**Mitophagy biomarkers**	**N = 200**	**N = 100**	**N = 100**	**N = 46**			
CSF PINK1 ng/mL	1.2 (0.2)	1.3 (0.2)	1.1 (0.2)	1.0 (0.3)	**<0.001**	0.09	**<0.001**
Serum PINK1, ng/mL	4.8 (10.7)	6.4 (13.3)	3.2 (6.8)	5.1 (10.1)	**0.01**	0.21	0.73
Serum BNIP3L, ng/mL	2.5 (1.8)	2.9 (2.1)	2.1 (1.2)	2.7 (2.2)	**0.02**	0.48	0.60
Serum TFEB, pg/mL	705.0 (361.8)	670.1 (368.6)	744.3 (351.9)	966.5 (1562.5)	**0.03**	0.90	**0.04**
**AD biomarkers**							
CSF Aβ42/40	0.04 (0.02)	0.04 (0.01)	0.04 (0.02)	0.09 (0.02)	0.74	**<0.001**	**<0.001**
CSF Aβ42, pg/mL	486.5 (187.2)	493.0 (203.2)	480.0 (170.5)	1138.8 (474.7)	0.99	**<0.001**	**<0.001**
CSF t-MAPT/tau, pg/mL	618.8 (409.2)	667.3 (465.0)	570.9 (341.3)	225.5 (113.1)	0.17	**<0.001**	**<0.001**
CSF p-MAPT/tau (181), pg/mL	101.9 (65.7)	107.4 (69.6)	96.4 (61.5)	37.2 (17.6)	0.29	**<0.001**	**<0.001**
Serum NEFL pg/mL	35.342 (26.4)	38.7 (21.0)	35.8 (31.2)	14.0 (5.6)	0.10	**<0.001**	**<0.001**
CSF NEFL pg/mL	1293.0 (910.3)	1571.2 (979.8)	1304.2 (864.1)	666.1 (455.3)	0.04	**<0.001**	**<0.001**
CSF NRGN, pg/mL	253.7 (103.8)	267.3 (107.8)	253.8 (101.6)	188.2 (70.8)	0.77	**0.03**	**0.01**

Data are presented as N (%) and mean (SD) unless otherwise specified.

*P* values are comparisons using Tukey post hoc tests (one-way analysis of covariance was used to test the main between group differences), ^a^data were log-tranformed.

Abbreviations: MCI, mild cognitive impairment; AD, Alzheimer’s disease; CU, cognitively unimpaired; CSF, cerebrospinal fluid; NEFL, neurofilament light chain; NRGN, neurogranin.

**Table 1.** Characteristic of study participants.

	Memory Clinic Cohort				P-values		
	All patients	AD Dementia	AD-MCI Patients	CU Controls	AD-MCI vs. ADDementia	AD MCIvs. CU	AD Dementiavs. CU
**Demographic characteristics**	**N = 200**	**N = 100**	**N = 100**	**N = 46**			
Age, years	71.4 (7.9)	70.3 (8.6)	72.5 (6.9)	63.9 (9.4)	0.16	**<0.001**	**<0.001**
Female, n (%)	121 (60.5)	64 (64.0)	57 (57.0)	30 (65.2)	0.31	0.11	0.39
Education, years	14.4 (3.0)	14.0 (2.9)	14.7 (3.1)	15.6 (2.9)	0.16	0.29	**0.01**
*APOE* ɛ4 positive, n (%)*	110 (55.0)	52 (52.0)	58 (58.0)	5 (10.9)	0.78	**<0.001**	**<0.001**
MMSE score	22.0 (4.8)	18.5 (4.2)	24.9 (3.0)	29.0 (1.3)	**<0.001**	**<0.001**	**<0.001**
GDS-15	3.3 (2.7)	3.6 (3.1)	3.0 (2.4)	2.2 (2.2)	0.43	0.29	**0.04**
BAI	8.8 (7.6)	9.11 (7.5)	8.6 (7.6)	9.3 (6.6)	0.91	0.92	0.99
Comorbidities**							
Hypertension, n (%)	89 (44.5)	41 (41.0)	48 (48.0)	17 (36.9)	0.78	0.89	0.99
Hypercholesterolemia, n (%)	66 (33.0)	32 (32.0)	34 (34.0)	13 (28.2)	0.99	0.99	0.99
Ischemic heart disease, n (%)	12 (6.0)	6 (6.0)	6 (6.0)	0 (0)	0.99	0.31	0.28
Chronic obstructive pulmonary disease, n (%)	14 (7.0)	5 (5.0)	9 (9.0)	1 (2.2)	0.52	0.32	0.82
Thyroid disease, n (%)	37 (18.5)	16 (16.0)	21 (21.0)	10 (21.7)	0.83	0.80	0.52
**Neuropsychological tests**							
DS–F^a^	–	7.2 (2.2)	8.4 (2.0)	9.4 (2.4)	**0.002**	**0.05**	**<0.001**
DS–B^a^	–	3.7 (1.7)	5.1 (1.7)	6.6 (1.7)	**<0.001**	**<0.001**	**<0.001**
TMT A^a^	–	107.1 (56.0)	64.4 (32.3)	37.2 (13.1)	**<0.001**	**0.002**	**<0.001**
TMT B^a^	–	272.3 (61.9)	198.8 (86.5)	103.6 (60.7)	**<0.001**	**<0.001**	**<0.001**
p–VF^a^	–	25.1 (10.3)	37.2 (13.4)	47.4 (12.7)	**<0.001**	**<0.001**	**<0.001**
S–VF^a^	–	11.6 (5.2)	16.5 (5–3)	27.2 (6.2)	**<0.001**	**<0.001**	**<0.001**
BNT^a^	–	9.9 (5.0)	6.2 (4.5)	1.7 (1.7)	**<0.001**	**<0.001**	**<0.001**
CDT^a^	–	9.4 (3.9)	13.0 (2.4)	15.5 (0.7)	**<0.001**	**0.003**	**<0.001**
ROCF–C^a^	–	18.0 (10.5)	25.3 (6.6)	30.0 (3.3)	**<0.001**	**0.007**	**<0.001**
LM–IR^a^	–	4.6 (3.4)	8.2 (4.2)	18.0 (4.4)	**<0.001**	**<0.001**	**<0.001**
LM–DR^a^	–	1.6 (2.4)	3.7 (4.6)	15.8 (5.4)	**0.005**	**<0.001**	**<0.001**
**Brain structures**							
Hippocampal volume (mm^3^)	–	6625.1 (862.7)	6692.84 (847.5)	7753.0 (755.9)	0.91	**<0.001**	**<0.001**
Entorhinal cortical thickness (mm)	–	2.6 (0.4)	2.7 (0.3)	3.0 (0.4)	0.42	**<0.001**	**<0.001**
Parahippocampal cortical thickness (mm)	–	2.3 (0.3)	2.4 (0.2)	2.6 (0.2)	0.06	**0.004**	**<0.001**
Gray matter volume (mm^3^)	–	540783.2 (44811.5)	559119.6 (52236.7)	594469.5 (31371.8)	0.12	**0.002**	**<0.001**
Ventricular volume (mm^3^)	–	49033.5 (17036.1)	47526.6 (19204.2)	41637.7 (29888.3)	0.93	0.43	0.33

Data are presented as N (%) and mean (SD) unless otherwise specified.

*P* values are comparisons using Tukey post hoc tests (one-way analysis of variance was used to test the main between group differences).

Abbreviations: MCI, mild cognitive impairment; AD, Alzheimer disease; CU, cognitively unimpaired; APOE, apolipoprotein E; MMSE, Mini Mental State Examination; GDS-15, Geriatric Depression Scale, 15-item version; BAI, Beck Anxiety Inventory; DS-F, Digit Span Forward; DS-B, Digit Span Backward; TMT, Trail Making Test; p-VF, phonemic verbal fluency (letters N, K, P); S-VF, semantic verbal fluency (animals); BNT, Boston Naming Test (30 odd-items version), number of errors; CDT, Clock Drawing Test; ROCF-C, Rey-Osterrieth Complex Figure, copy condition; LM-IR, Logical Memory Story I, immediate recall; LM-DR, Logical Memory Story I, delayed recall.

*Missing genotype data in *n* = 35.

**Missing data in AD dementia (*n* = 5), AD MCI (*n* = 1), CU controls (*n* = 6).

Within the AD dementia and MCI-AD group, there were 110 (55%) *APOE* ε4 carriers: (84 *APOE* ɛ4 heterozygotes and 26 *APOE* ɛ4 homozygotes). There were five (10.9%) *APOE* ε4 heterozygotes and no homozygotes in the CU group.

Besides the clinical classification, all the participants undergoing CSF collection (*n* = 218) were also classified according to the AT(N) criteria framework. To define the AT(N) status, we used CSF Aβ42 as “A”, CSF p-MAPT/tau (181) as “T”, and CSF t-MAPT/tau as “N” [48]. These biomarkers were dichotomized as normal (-) or abnormal (+), and the patients were divided into four groups: A-T-N- (no pathology, *n* = 36), A+T-N- (amyloid pathology, *n* = 38), A+T+N- (AD pathology, *n* = 33), A+T+N+ (AD pathology with neurodegeneration, *n* = 104) [16].

### Exclusion criteria

Participants with moderate to severe white matter vascular lesions on MRI (Fazekas score > 2 points) or any primary neurological or psychiatric disorders that could cause cognitive or mitochondrial dysfunction (*e.g*., major depressive disorder, psychosis, Parkinson disease, Lewy body dementia, frontotemporal lobar degeneration, substance abuse) were excluded from the study. Patients with evidence of cancer, diabetes, renal failure, cardiac failure, gait disorders, parkinsonian syndromes, hemiparesis, or any neurological disease were also excluded from the study.

### CSF and blood collection and processing

Blood samples were drawn by venipuncture, allowed to clot at room temperature for 15 min, and centrifuged at 1700 × g at 20°C for 5 min within 30 min of collection. Serum supernatant was collected, divided into 0.5 ml polypropylene aliquots, and stored at − 80°C until further use.

CSF was obtained by lumbar puncture in a supine position at L3/L4 or L4/L5, collected in 8-mL polypropylene tubes, gently mixed, and centrifuged at 1700 × g at 20°C for 5 min within 30 min of collection. CSF was aliquoted in polypropylene tubes of 0.5 ml and stored at −80°C until analysis. Before biomarker analysis, serum and CSF samples stored at − 80°C were thawed and vortexed for 15 seconds.

### Immunological assays

A LUMIPULSE® G600II instrument (Fujirebio, Ghent, Belgium) was used to measure CSF levels of Aβ42, Aβ40, t-MAPT/tau, p-MAPT/tau (181), and NRGN. In this cartridge-based system, monoclonal antibody-coated beads are used for capture, and monoclonal antibodies are used for detection. Luminiscence was measured at 477 nm [44].

Commercial enzyme-linked immunosorbent assay (ELISA; UmanDiagnostics, 10-7001CE) was used to measure CSF levels of NEFL. Protein levels of mitophagy biomarkers – PINK1 in CSF and serum and BNIP3L and TFEB in serum – were also quantified using commercially available ELISA kits (FineTest, EH6731 for BNIP3L; MyBioSource, MBS7607221 for PINK1 and MBS7612687 for TFEB) following the manufacturer’s instructions. Serum and CSF samples were measured in duplicate. At the end of the assay, absorbances were read at 450 nm using a microplate reader (Dynex Technologies, Virginia, USA), and the protein concentration was calculated by comparison with a standard curve. The NEFL ELISA kit stated an intra-assay coefficient of variance (CV%) of less than 8% and an inter-assay CV < 10%. The BNIP3L, PINK1 and TFEB ELISA kits stated an intra-assay CV < 5% and an inter-assay CV < 10%.

### Neuropsychological assessment

Cognitive performance was assessed using the following tests: (a) global cognitive function measured with Mini-Mental State Examination/MMSE [49]; (b) attention and working memory measured with the Forward/DS-F and Backward/DS-B Digit Span subtests (from the Wechsler Adults Intelligence Scale – Revised), and the Trail Making Test (TMT) A [50]; (c) memory measured with the Logical Memory/LM immediate and delayed recall, an adaptation from the Uniform Data Set (UDS-cz 2.0) [51]; (d) language measured with the Boston Naming Test (BNT-30), 30 odd-items version [52], and semantic verbal fluency/S-VF, animals [53]; (e) executive function measured with TMT B [50], and phonemic verbal fluency/P-VF (Czech version with letters N, K, P) [53]; and (f) visuospatial function measured with the Rey-Osterrieth Complex Figure Task/ROCFT – the copy condition, and the Clock Drawing Test/CDT [54]. The self-report Geriatric Depression Scale/GDS-15, a 15-item version [55], and the Beck Anxiety Inventory/BAI [56] were administered to evaluate anxiety and depressive symptoms. Mean cognitive performance values (±SD) are listed for each patient subgroup in Table 1.

### MRI acquisition and analysis

MRI images were acquired on a 1.5T scanner (Siemens, Erlangen, Germany) using T1-weighted three-dimensional high-resolution magnetization-prepared rapid acquisition with gradient echo sequence using the following parameters: TR/TE/TI = 2000/3.08/1100 ms, flip angle 15°, 192 continuous partitions, slice thickness 1.0 mm, and in-plane resolution 1 mm [57]. All images were inspected visually by a neuroradiologist in a blinded manner. Patients whose MRI data showed evidence of tumor, cortical infarct, hydrocephalus, or other major anatomical variation were excluded from the study. Freesurfer automated suite (v7.1.0, http://surfer.nmr.mgh.harvard.edu) was used to derived regional cortical thickness [58], subcortical areas and volumes [59], as well as total hippocampal volume, entorhinal cortical thickness, parahippocampal cortical thickness, total gray matter volume, and ventricular volumes. Brain area and volume measurements were normalized to the estimated total intracranial volume (eTIV [60],) as follows: Vol_adj_=Vol_raw_−β(TIV_raw_−TIV_mean_); regional thickness measurements were not eTIV-adjusted [61]. Morphometric characteristics of the participants are listed in Table 1.

### Statistical analysis

All data were standardized to z-scores. Data with non-normal distribution were log-transformed (mitophagy markers, AD biomarkers, hippocampal volume, ventricle volumes) prior to transformation to z-scores.

Between-group differences in demographical characteristics were evaluated using parametric one-way analysis of variance (ANOVA) with Tukey post hoc tests for continuous variables (age, years of education, Mini-Mental State Examination, Geriatric Depression Scale and Beck Anxiety Inventory score) and chi-square tests for dichotomous variables (sex, *APOE* ɛ4 status).

Between-group differences in mitophagy markers were evaluated using parametric one-way analysis of covariance (ANCOVA), with Tukey post hoc test. Each ANCOVA model included the mean value of the mitophagy biomarker level as the outcome, the study group as a between-subject factor, and covariates of age and sex.

The relationships between mitophagy markers (PINK1, BNIP3L, TFEB), AD biomarkers (Aβ42/40, Aβ42, p-MAPT/tau [181]), t-MAPT/tau, NEFL, NRGN), AT(N) profiles and AD-related structures (hippocampal volume, gray matter volume, ventricle volumes, entorhinal thickness, parahippocampal thickness) were evaluated using partial Pearson correlation adjusted for age and sex. Pearson correlation adjusted for age, sex, and years of education were used to establish the relationship between mitophagy markers (PINK1, BNIP3L, TFEB) with the cognitive composite domain (attention and working memory, memory, executive function, language, and visuospatial function). Holm-Bonferroni correction was used to adjust for multiple comparisons.

Cognitive domains for the participants with AD-dementia and AD-MCI were expressed as composite domain z-scores, calculated as the average of z-scores for each of the tests within the specific cognitive domain. The z-scores for TMT A and B, and Boston Naming Test-30 were multiplied by negative ones to express the values in the same direction as the other neuropsychological values. The maximum time for completion of the TMT A and B were 180 s and 300 s, respectively, and those who were unable to complete the tests were assigned a score of as 181 s and 301 s, respectively.

*p*-values <0.05 (*), 0.01 (**), and 0.001 (***) were considered statistically significant. Analyses were performed using the R statistical language environment [62].

## References

[1] Fang EF, Xie C, Schenkel JA, et al. A research agenda for ageing in China in the 21st century (2nd edition): Focusing on basic and translational research, long-term care, policy and social networks. Ageing Res Rev. 2020;64:101174. doi: 10.1016/J.ARR.2020.101174 32971255 PMC7505078

[2] Hou Y, Dan X, Babbar M, et al. Ageing as a risk factor for neurodegenerative disease. Nat Rev Neurol. 2019;15:565–581. doi: 10.1038/S41582-019-0244-7 31501588

[3] Mintun MA, Lo AC, Evans CD, Wessels AM, Ardayfio PA, Andersen SW, et al. Donanemab in early Alzheimer’s disease. New England Journal of Medicine 2021;384:1691–704. doi: 10.1056/NEJMOA2100708 33720637

[4] van Dyck C, Swanson C, Aisen P, et al. Lecanemab in early alzheimer’s disease. N Engl J Med. 2023;388(1):9–21. doi: 10.1056/NEJMC2301380 36449413

[5] Lautrup S, Sinclair DA, Mattson MP, et al. NAD+ in brain aging and neurodegenerative disorders. Cell Metab. 2019;30:630. doi: 10.1016/J.CMET.2019.09.001 31577933 PMC6787556

[6] Aman Y, Schmauck-Medina T, Hansen M, et al. Autophagy in healthy aging and disease. Nat Aging. 2021;1:634–650. doi: 10.1038/S43587-021-00098-4 34901876 PMC8659158

[7] Rubinsztein DC, Mariño G, Kroemer G. Autophagy and aging. Cell. 2011;146:682–695. doi: 10.1016/J.CELL.2011.07.030 21884931

[8] Sun N, Youle RJ, Finkel T. The mitochondrial basis of aging. Mol Cell. 2016;61:654–666. doi: 10.1016/J.MOLCEL.2016.01.028 26942670 PMC4779179

[9] Palikaras K, Lionaki E, Tavernarakis N. Mechanisms of mitophagy in cellular homeostasis, physiology and pathology. Nat Cell Biol. 2018;20:1013–1022. doi: 10.1038/S41556-018-0176-2 30154567

[10] Lou G, Palikaras K, Lautrup S, et al. Mitophagy and neuroprotection. Trends Mol Med. 2020;26:8–20. doi: 10.1016/J.MOLMED.2019.07.002 31375365

[11] Xie C, Zhuang XX, Niu Z, et al. Amelioration of Alzheimer’s disease pathology by mitophagy inducers identified via machine learning and a cross-species workflow. Nat Biomed Eng. 2022;6:76. doi: 10.1038/S41551-021-00819-5 34992270 PMC8782726

[12] Fang EF, Hou Y, Palikaras K, et al. Mitophagy inhibits amyloid-β and tau pathology and reverses cognitive deficits in models of Alzheimer’s disease. Nat neurosci. 2019;22(3):401–412. doi: 10.1038/s41593-018-0332-9 30742114 PMC6693625

[13] Kerr JS, Adriaanse BA, Greig NH, et al. Mitophagy and Alzheimer’s disease: cellular and molecular mechanisms. Trends Neurosci. 2017;40:151. doi: 10.1016/J.TINS.2017.01.002 28190529 PMC5341618

[14] Menzies FM, Fleming A, Caricasole A, et al. Autophagy and neurodegeneration: pathogenic mechanisms and therapeutic opportunities. Neuron. 2017;93(5):1015–1034. doi: 10.1016/J.NEURON.2017.01.022/ATTACHMENT/9A584587-3E18-4AF0-B0AF-D037F4A3B294/MMC1.PDF 28279350

[15] Caponio D, Veverová K, Zhang S, et al. Compromised autophagy and mitophagy in brain ageing and Alzheimer’s diseases. Aging Brain. 2022;2:100056. doi: 10.1016/J.NBAS.2022.100056 36908880 PMC9997167

[16] Du F, Yu Q, Yan S, et al. PINK1 signalling rescues amyloid pathology and mitochondrial dysfunction in Alzheimer’s disease. Brain. 2017;140:3233. doi: 10.1093/BRAIN/AWX258 29077793 PMC5841141

[17] Shi L, Baird AL, Westwood S, et al. A decade of blood biomarkers for alzheimer’s disease research: An evolving field, improving study designs, and the Challenge of Replication. J Alzheimers Dis. 2018;62(3):1181–1198. doi: 10.3233/JAD-170531 29562526 PMC5870012

[18] Nixon RA, Wegiel J, Kumar A, et al. Extensive involvement of autophagy in Alzheimer disease: an immuno-electron microscopy study. J Neuropathol Exp Neurol. 2005;64:113–122. doi: 10.1093/JNEN/64.2.113 15751225

[19] Yu W H, Cuervo AM, Kumar A, et al. Macroautophagy—a novel β-amyloid peptide-generating pathway activated in Alzheimer’s disease. The Journal Of Cell Biology. 2005;171(1):87. doi: 10.1083/JCB.200505082 16203860 PMC2171227

[20] Martín-Maestro P, Gargini R, Perry G, et al. PARK2 enhancement is able to compensate mitophagy alterations found in sporadic Alzheimer’s disease. Hum Mol Genet. 2016;25(4):792. doi: 10.1093/HMG/DDV616 26721933 PMC4743695

[21] Ye X, Sun X, Starovoytov V, et al. Parkin-mediated mitophagy in mutant hAPP neurons and Alzheimer’s disease patient brains. Human Molecular Genetics. 2015;24(10):2938. doi: 10.1093/HMG/DDV056 25678552 PMC4406302

[22] Hou X, Watzlawik JO, Cook C, et al. Mitophagy alterations in Alzheimer’s disease are associated with granulovacuolar degeneration and early tau pathology. Alzheimer’s & Dementia. 2021;17(3):417. doi: 10.1002/ALZ.12198 PMC8048674 33090691

[23] Sohn HY, Kim SI, Park JY, et al. ApoE4 attenuates autophagy via FoxO3a repression in the brain. Sci Rep. 2021;11(1):1–10. doi: 10.1038/s41598-021-97117-6 34475505 PMC8413297

[24] Vaillant-Beuchot L, Mary A, Pardossi-Piquard R, et al. Accumulation of amyloid precursor protein C-terminal fragments triggers mitochondrial structure, function, and mitophagy defects in Alzheimer’s disease models and human brains. Acta Neuropathol. 2021;141:39–65. doi: 10.1007/S00401-020-02234-7 33079262 PMC7785558

[25] Bordi M, Berg MJ, Mohan PS, et al. Autophagy flux in CA1 neurons of Alzheimer hippocampus: increased induction overburdens failing lysosomes to propel neuritic dystrophy. Autophagy. 2016;12:2467. doi: 10.1080/15548627.2016.1239003 27813694 PMC5173282

[26] Qian S, He H, Xiong X, et al. Identification of mitophagy-associated proteins profile as potential plasma biomarkers of idiopathic Parkinson’s disease. CNS Neurosci Ther. 2023. doi: 10.1111/CNS.14532 PMC11056850 37990436

[27] Cossu D, Yokoyama K, Sechi LA, et al. Potential of PINK1 and PARKIN proteins as biomarkers for active multiple sclerosis: a Japanese cohort study. Front Immunol. 2021;12. doi: 10.3389/FIMMU.2021.681386 PMC8371632 34421896

[28] Armstrong A, Mattsson N, Appelqvist H, et al. Lysosomal network proteins as potential novel CSF biomarkers for Alzheimer’s disease. NeuroMol Med. 2014;16(1):150–160. doi: 10.1007/S12017-013-8269-3 PMC3918123 24101586

[29] Sjödin S, Brinkmalm G, Öhrfelt A, et al. Endo-lysosomal proteins and ubiquitin CSF concentrations in Alzheimer’s and Parkinson’s disease. Alzheimers Res Ther. 2019;11(1):11. doi: 10.1186/S13195-019-0533-9 31521194 PMC6745076

[30] Eskelinen EL. Roles of LAMP-1 and LAMP-2 in lysosome biogenesis and autophagy. Mol Aspects Med. 2006;27:495–502. doi: 10.1016/j.mam.2006.08.005 16973206

[31] Menzies FM, Moreau K, Puri C, et al. Measurement of autophagic activity in mammalian cells. Curr Protoc Cell Biol. 2012;54:.15.16.1–.15.16.25. doi: 10.1002/0471143030.CB1516S54 22422474

[32] Klionsky DJ, Abdel-Aziz AK, Abdelfatah S, et al. Guidelines for the use and interpretation of assays for monitoring autophagy (4th edition)1. Autophagy. 2021;17:1–382. DOI:10.1080/15548627.2020.1797280 PMC7996087 33634751

[33] Sardiello M, Palmieri M, Di RA, et al. A gene network regulating lysosomal biogenesis and function. Science. 2009;325:473–477. doi: 10.1126/SCIENCE.1174447 19556463

[34] Tiribuzi R, Crispoltoni L, Porcellati S, et al. miR128 up-regulation correlates with impaired amyloid β(1-42) degradation in monocytes from patients with sporadic Alzheimer’s disease. Neurobiology Of Aging. 2014;35(2):345–356. doi: 10.1016/J.NEUROBIOLAGING.2013.08.003 24064186

[35] Wang H, Wang R, Xu S, et al. Transcription factor eb is selectively reduced in the nuclear fractions of alzheimer’s and amyotrophic lateral sclerosis brains. Neurosci J. 2016;2016:1–8. doi: 10.1155/2016/4732837 PMC4940567 27433468

[36] Martín-Maestro P, Sproul A, Martinez H, et al. Autophagy induction by Bexarotene promotes mitophagy in Presenilin 1 familial Alzheimer’s disease iPSC-derived neural stem cells. Mol Neurobiol. 2019;56:8220. doi: 10.1007/S12035-019-01665-Y 31203573 PMC6842097

[37] Sass F, Schlein C, Jaeckstein MY, et al. TFEB deficiency attenuates mitochondrial degradation upon brown adipose tissue whitening at thermoneutrality. Mol Metab. 2021;47:101173. doi: 10.1016/J.MOLMET.2021.101173 33516944 PMC7903014

[38] Song JX, Malampati S, Zeng Y, et al. A small molecule transcription factor EB activator ameliorates beta‐amyloid precursor protein and Tau pathology in Alzheimer’s disease models. Aging Cell. 2020 19;19(2). doi: 10.1111/ACEL.13069 PMC6996953 31858697

[39] Xiao Q, Yan P, Ma X, et al. Neuronal-targeted TFEB accelerates lysosomal degradation of app, reducing Aβ generation and amyloid plaque pathogenesis. J Neurosci. 2015;35:12137. doi: 10.1523/JNEUROSCI.0705-15.2015 26338325 PMC4556784

[40] Zhang YD, Zhao JJ. TFEB Participates in the Aβ-induced pathogenesis of alzheimer’s disease by regulating the autophagy-lysosome pathway. DNA Cell Biol. 2015;34(11):661–668. doi: 10.1089/DNA.2014.2738 26368054

[41] Jack CR, Knopman DS, Jagust WJ, et al. Tracking pathophysiological processes in Alzheimer’s disease: an updated hypothetical model of dynamic biomarkers. The Lancet Neurology. 2013;12(2):207–216. doi: 10.1016/S1474-4422(12)70291-0 23332364 PMC3622225

[42] Glatigny M, Moriceau S, Rivagorda M, et al. Autophagy is required for memory formation and reverses age-related memory decline. Curr Biol. 2019;29(3):435–448.e8. doi: 10.1016/J.CUB.2018.12.021 30661803

[43] McKhann GM, Knopman DS, Chertkow H, et al. The diagnosis of dementia due to Alzheimer’s disease: recommendations from the national institute on aging-alzheimer’s association workgroups on diagnostic guidelines for Alzheimer’s disease. Alzheimer’s & Dementia. 2011;7(3):263. doi: 10.1016/J.JALZ.2011.03.005 PMC3312024 21514250

[44] Gobom J, Parnetti L, Rosa-Neto P, et al. Validation of the LUMIPULSE automated immunoassay for the measurement of core AD biomarkers in cerebrospinal fluid. Clin Chem Lab Med. 2022;60(2):207–219. doi: 10.1515/CCLM-2021-0651/MACHINEREADABLECITATION/RIS 34773730

[45] Scheltens P, Kuiper M, Ch Wolters E, et al. Atrophy of medial temporal lobes on MRI in probable Alzheimer’s disease and normal ageing: diagnostic value and neuropsychological correlates. Journal of Neurology, Neurosurgery & Psychiatry. 1992;55(10):967–972. doi: 10.1136/JNNP.55.10.967 1431963 PMC1015202

[46] Albert MS, DeKosky ST, Dickson D, et al. The diagnosis of mild cognitive impairment due to Alzheimer’s disease: recommendations from the national institute on aging-alzheimer’s association workgroups on diagnostic guidelines for Alzheimer’s disease. Alzheimer’s & Dementia. 2011;7(3):270. doi: 10.1016/J.JALZ.2011.03.008 PMC3312027 21514249

[47] Jessen F, Amariglio RE, Van Boxtel M, et al. A conceptual framework for research on subjective cognitive decline in preclinical Alzheimer’s disease. Alzheimer’s & Dementia. 2014;10(6):844. doi: 10.1016/J.JALZ.2014.01.001 PMC4317324 24798886

[48] Jack CR, Bennett DA, Blennow K, et al. NIA-AA research framework: toward a biological definition of Alzheimer’s disease. Alzheimer’s & Dementia. 2018;14(4):535. doi: 10.1016/J.JALZ.2018.02.018 PMC5958625 29653606

[49] Štěpánková H, Nikolai T, Lukavský J, et al. Mini-mental state examination—Czech normative study. Ceska a Slovenska Neurologie a Neurochirurgie. 2015;78(3):292–299. doi: 10.14735/amcsnn201557

[50] Bezdicek O, Motak L, Axelrod BN, et al. Czech version of the trail making test: normative data and clinical utility. Arch Clin Neuropsychol. 2012;27:906–914. doi: 10.1093/ARCLIN/ACS084 23027441

[51] Nikolai T, Stepankova H, Kopecek M, et al. The uniform data set, czech version: normative data in older adults from an international perspective. J Alzheimers Dis. 2018;61(3):1233. doi: 10.3233/JAD-170595 29332045 PMC6939612

[52] Bezdicek O, Rosická AM, Mana J, et al. The 30-item and 15-item boston naming test czech version: item response analysis and normative values for healthy older adults. J Clin Exp Neuropsychol. 2022;43(9):890–905. doi: 10.1080/13803395.2022.2029360 35125051

[53] Nikolai T, Štěpánková H, Michalec J, et al. Verbal fluency tests—Czech normative study for older persons. Ceska a Slovenska Neurologie a Neurochirurgie. 2015;78(3):292–299. doi: 10.14735/amcsnn2015292

[54] Mazancova AF, Nikolai T, Stepankova H, et al. The reliability of clock drawing test scoring systems modeled on the normative data in healthy aging and nonamnestic mild cognitive impairment. Assessment. 2017;24:945–957. doi: 10.1177/1073191116632586 26933141

[55] Sheikh JI, Yesavage JA. Geriatric depression scale (gds): recent evidence and development of a shorter version. Clin Gerontologist. 1986;5(1–2):165–173. doi: 10.1300/J018V05N01_09

[56] Beck AT, Epstein N, Brown G, et al. An inventory for measuring clinical anxiety: psychometric properties. J Consult Clin Psychol. 1988;56:893–897. doi: 10.1037/0022-006X.56.6.893 3204199

[57] Amlerova J, Laczó J, Nedelska Z, et al. Emotional prosody recognition is impaired in Alzheimer’s disease. Alzheimers Res Ther. 2022;14(1):14. doi: 10.1186/S13195-022-00989-7 35382868 PMC8985328

[58] Fischl B, Van Der Kouwe A, Destrieux C, Halgren E, Ségonne F, Salat DH, et al. Automatically parcellating the human cerebral cortex. Cerebral Cortex. 2004;14:11–22. doi: 10.1093/CERCOR/BHG087 14654453

[59] Fischl B, Salat DH, Busa E, et al. Whole brain segmentation: automated labeling of neuroanatomical structures in the human brain. Neuron. 2002;33:341–355. doi: 10.1016/S0896-6273(02)00569-X 11832223

[60] Voevodskaya O, Simmons A, Nordenskjöld R, et al. The effects of intracranial volume adjustment approaches on multiple regional MRI volumes in healthy aging and Alzheimer’s disease. Front Aging Neurosci. 2014;6:264. doi: 10.3389/FNAGI.2014.00264/BIBTEX 25339897 PMC4188138

[61] Schwarz CG, Gunter JL, Wiste HJ, et al. A large-scale comparison of cortical thickness and volume methods for measuring Alzheimer’s disease severity. NeuroImage Clin. 2016;11:802. doi: 10.1016/J.NICL.2016.05.017 28050342 PMC5187496

[62] RStudio Team. Rstudio: integrated Development for R 2020.

